# Randomized trial on the effects of a combined physical/cognitive training in aged MCI subjects: the Train the Brain study

**DOI:** 10.1038/srep39471

**Published:** 2017-01-03

**Authors:** Train the Brain Consortium, L. Maffei, E. Picano, M. G. Andreassi, A. Angelucci, F. Baldacci, L. Baroncelli, T. Begenisic, P. F. Bellinvia, N. Berardi, L. Biagi, J. Bonaccorsi, E. Bonanni, U. Bonuccelli, A. Borghini, C. Braschi, M. Broccardi, R. M. Bruno, M. Caleo, C. Carlesi, L. Carnicelli, G. Cartoni, L. Cecchetti, M. C. Cenni, R. Ceravolo, L. Chico, S. Cintoli, G. Cioni, M. Coscia, M. Costa, G. D’Angelo, P. D’Ascanio, M. De Nes, S. Del Turco, E. Di Coscio, M. Di Galante, N. di Lascio, F. Faita, I. Falorni, U. Faraguna, A. Fenu, L. Fortunato, R. Franco, L. Gargani, R. Gargiulo, L. Ghiadoni, F. S. Giorgi, R. Iannarella, C. Iofrida, C. Kusmic, F. Limongi, M. Maestri, M. Maffei, S. Maggi, M. Mainardi, L. Mammana, A. Marabotti, V. Mariotti, E. Melissari, A. Mercuri, S. Micera, S. Molinaro, R. Narducci, T. Navarra, M. Noale, C. Pagni, S. Palumbo, R. Pasquariello, S. Pellegrini, P. Pietrini, T. Pizzorusso, A. Poli, L. Pratali, A. Retico, E. Ricciardi, G. Rota, A. Sale, S. Sbrana, G. Scabia, M. Scali, D. Scelfo, R. Sicari, G. Siciliano, F. Stea, S. Taddei, G. Tognoni, A. Tonacci, M. Tosetti, S. Turchi, L. Volpi

**Affiliations:** 1 Institute of Neuroscience of the CNR, Via G. Moruzzi 1, 56100 Pisa, Italy; 2 Institute of Clinical Physiology of the CNR, Via G. Moruzzi 1, 56124 Pisa, Italy; 3 Department of Clinical and Experimental Medicine-Neurology Unit, University of Pisa & AOU Pisa, Italy; 4 IRCCS Stella Maris, Viale del Tirreno 341, Calambrone, Italy; 5 Department of Surgical, Medical, Molecular Pathology and of Critical Care, University of Pisa, Via Savi 10, 56126, Pisa, Italy; 6 Department of Clinical and Experimental Medicine, Pisa University, Via Savi 10, 56126 Pisa, Italy; 7 Department of Translational research and New technologies in Medicine and Surgery, Pisa University, Via Savi 10, 56126 Pisa, Italy; 8 Bertarelli Foundation Chair in Translational Neuroengineering, Center for Neuroprosthetics and Institute of Bioengineering, Ecole Polytechnique Federale de Lausanne, CH-1015 Lausanne, Switzerland; 9 Scuola Superiore Sant’Anna, P.za Martiri della Libertà 33, 56127 Pisa, Italy; 10 National Institute of Nuclear Physics (INFN), Pisa Section, Largo B. Pontecorvo, 3 56127, Pisa, Italy

## Abstract

Age-related cognitive impairment and dementia are an increasing societal burden. Epidemiological studies indicate that lifestyle factors, e.g. physical, cognitive and social activities, correlate with reduced dementia risk; moreover, positive effects on cognition of physical/cognitive training have been found in cognitively unimpaired elders. Less is known about effectiveness and action mechanisms of physical/cognitive training in elders already suffering from Mild Cognitive Impairment (MCI), a population at high risk for dementia. We assessed in 113 MCI subjects aged 65–89 years, the efficacy of combined physical-cognitive training on cognitive decline, Gray Matter (GM) volume loss and Cerebral Blood Flow (CBF) in hippocampus and parahippocampal areas, and on brain-blood-oxygenation-level-dependent (BOLD) activity elicited by a cognitive task, measured by ADAS-Cog scale, Magnetic Resonance Imaging (MRI), Arterial Spin Labeling (ASL) and fMRI, respectively, before and after 7 months of training vs. usual life. Cognitive status significantly decreased in MCI-no training and significantly increased in MCI-training subjects; training increased parahippocampal CBF, but no effect on GM volume loss was evident; BOLD activity increase, indicative of neural efficiency decline, was found only in MCI-no training subjects. These results show that a non pharmacological, multicomponent intervention improves cognitive status and indicators of brain health in MCI subjects.

The world is experiencing a substantial increase in the proportion of elderly adults in the population. With an aging society comes the increase in age related frailties, which may lead to cognitive impairments and to dementia, mostly in the form of Alzheimer’s Disease (AD). Age is indeed the major risk factor for dementia: the age-specific incidence rates for AD demonstrate a doubling of incidence for about every six years of added life^1^. The number of people with dementia worldwide was 35.6 million in 2010 and that is estimated to increase to 65.7 million by 2030 and 115.4 million by 2050 unless effective means of reducing the incidence of this disease are introduced^2^.

Several epidemiological studies have demonstrated that modifiable lifestyle factors, such as practicing physical exercise, being engaged in stimulating cognitive activities, maintaining an active social life into old age, and controlling nutrition, are correlated with maintaining good brain functioning, specifically in the elderly, and with reduced risk of developing dementia^3^,^4^,^5^,^6^,^7^,^8^. Norton *et al*.^9^ estimated that around one third of AD cases worldwide might be attributable to potentially modifiable risk factors which include several lifestyle habits. Interventions based on cognitive training^10^, diet^11^ or physical activity^7^,^12^,^13^ have indeed been found to produce positive effects on cognitive status in cognitively unimpaired adult or elderly subjects. Potential mechanisms underlying the effects of cognitive and physical activity on cognition, suggested also by studies in animal models, include increased hippocampal neurogenesis, increased Cerebral Blood Flow (CBF), and enhanced brain plasticity^14^,^15^.

An important point that has recently emerged both from the human and animal research, is that enhanced physical and cognitive activities seem to have additive effects on brain plasticity and age related cognitive decline^16^. In this line, a very important study is the FINGER trial^17^, in which 1260 subjects, aged 60–77 years, with cognition in the mean level for age and elevated Dementia Risk Score, were randomized to a 2 year multidomain intervention of diet, exercise, cognitive training, and vascular risk monitoring, showing that cognitive performances improved more in the intervention than in the control group.

Less is known about the effectiveness of exercise or cognitive training interventions on cognitive decline in older individuals already suffering from Mild Cognitive Impairment (MCI) or dementia and on the underlying mechanisms of action (see recent review papers^18^,^19^,^20^,^21^). MCI is characterized by objective deficits in one single domain (e.g. memory) or multiple domains of cognition, which do not yet configure as overt dementia^22^,^23^. MCI subjects are considered a population at high risk for dementia^24^,^25^,^26^,^27^,^28^,^29^, a large proportion of them proceeding indeed towards it. Therefore, they are a promising target of interventions aimed at reducing the incidence of dementia.

Here, we have undertaken a Randomized Control Study in a group of 113 MCI subjects to assess the efficacy of the combination of multidomain cognitive training and physical exercise in a social setting in reducing cognitive decline, in reducing Gray Matter (GM) volume loss, in increasing Cerebral Blood Flow (CBF) in hippocampus and parahippocampal areas, as well as in ameliorating task-related brain activity.

## Methods

### Study design and recruitment

**Figure 1:**
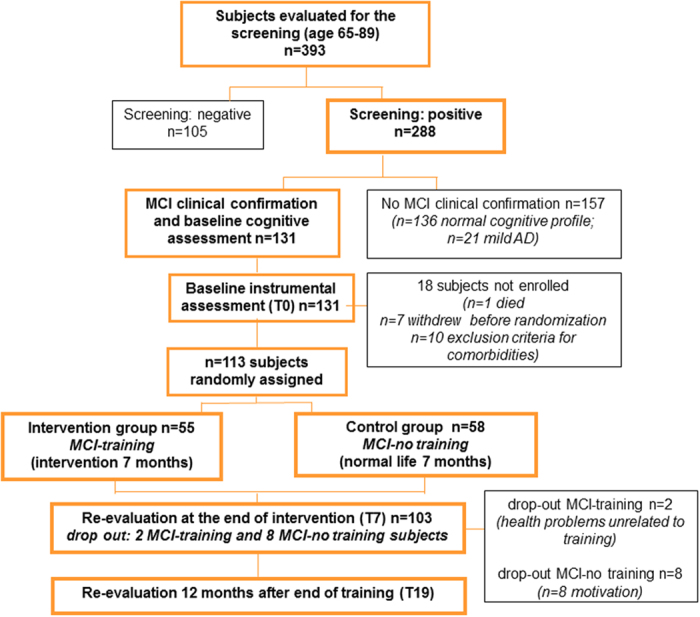
Flow chart of the study.

The flow chart of the study is reported in Fig. 1. The eligible population for the study included elderly subjects aged between 65 and 89 years with Mild Cognitive Impairment confirmed at the neurological examination according to the current guidelines.

Exclusion criteria were neurological pathologies, moderate or severe dementia (CDR > 1), clinical evidence for depression (GDS ≥ 9) or other psychiatric disorders, advanced neoplasia, severe aortic stenosis, obstructive hypertrophic cardiomiopathy, severe chronic renal insufficiency (glomerular filtration rate (GFR) < 35 ml/min), severe chronic obstructive pulmonary disease, uncompensated type II diabetes, arteriopathy class III Leriche-Fontaine, epilepsy, drug addiction, recent cranial trauma, sensory-motor deficits preventing participation to cognitive assessment and training or pathologies or orthopedic problems barring or limiting participation to the aerobic physical training program.

Subjects gave written informed consent for participation in the study. Study design and protocol, including subject privacy and sensitive data treatment, have been approved by the Regional Ethical Committee for Clinical Experimentation. ClinicalTrials.gov Identifier is NCT01725178 (November 8, 2012). All methods included in the protocol were carried out in accordance with the guidelines laid down in the Declaration of Helsinki.

Subject recruitment was performed using a two-phase scheme. A first screening phase was performed to select potentially eligible subjects from the general population. These potentially eligible subjects underwent a second phase of clinical confirmation, which also provided the baseline (T0) cognitive status. Subjects with a confirmed diagnosis of MCI and matching inclusion criteria underwent baseline instrumental evaluation.

Subjects have been recruited from the general population through several channels: General Practitioners of Pisa Municipality; network of medical centres such as Misericordia and Pubblica Assistenza ambulatories; self-referral of subjects and their relatives who received information on the study from newspapers, television programs, poster and leaflets set in pharmacies, GP practices, patient associations; Neurology Unit and Neurorehabilitation Unit of Pisa University (Azienda Ospedaliera Universitaria Pisa, AOUP) Santa Chiara Hospital, Pisa; Neurophysiopathology Unit Hospital “F. Lotti”, Pontedera. Preliminary questionnaire regarding memory disturbs was self-administered (MAC-Q)^30^.

Recruitment has been performed between January 2011 and May 2014.

#### Screening evaluation

Screening evaluation was performed with the following tests: Mini Mental State Examination (MMSE)^31^, largely used to screen for cognitive impairment and also used to estimate the severity of the same; Clock Drawing Test (CDT)^32^, a valid screening method for Mild Cognitive Impairment; Clinical Dementia Rating Scale (CDR)^33^, a structured interview with the patient and an informant, used to quantify the severity of symptoms of dementia; Geriatric Depression Scale (GDS)^34^, a self-report assessment used to identify depression in the elderly. A total of 393 subjects have been subjected to the screening phase, which was closed on May 2014 to meet the time schedule of the project.

To be eligible for the clinical confirmation phase, participants had to score ≥ 20 in MMSE and meet one of the two following operational criteria: i) CDR of 0.5, with at least a 0.5 in the memory domain, independently of CDT score; ii) CDT score lower or equal than 8, even if CDR equal 0.

As specified in the exclusion criteria, Geriatric Depression Scale (GDS) score had to be lower than 9 to allow inclusion.

One hundred and five out of 393 subjects did not meet these criteria and resulted negative to the screening; 288 resulted positive and went on to the second phase of clinical confirmation.

#### MCI clinical confirmation and baseline (T0) cognitive assessment

MCI is a heterogeneous clinical entity and it is a concept in constant evolution. We used the term MCI to refer to symptomatic predementia phase. MCI is a syndrome defined by clinical, cognitive and functional criteria. We recruited in the study subjects from the general population who displayed cognitive deficits in performing complex functional tasks. These subjects came to the screening phase because they had experienced a decrease in their cognitive performance with respect to the past; however, they maintained their independence of function in daily life, with minimal need for aid or assistance.

For the MCI diagnosis, the diagnostic criteria proposed by European Consortium on Alzheimer’s Disease Working Group on MCI^35^ were applied. A comprehensive battery of neuropsychological tasks was chosen to assess performance in several cognitive domains (see online additional Method section). Moreover, a questionnaire concerning demographic, occupational and social data, education, medical history, pharmacological drug use and lifestyle habits (tobacco smoking, alcohol consumption, recreational and physical activity) was filled in. A final clinical diagnosis had to be confirmed by the results of both the neuropsychological examination and the clinical evaluation.

Of the 288 subjects entered in the phase of clinical confirmation, 157 did not receive an MCI diagnosis (136 had a normal cognitive profile and 21 resulted sufficiently impaired, cognitively and functionally, to meet National Institute of Neurological and Communicative Disorders and Stroke/Alzheimer’s Disease and Related Disorders Association criteria for mild AD) and 131 received an MCI diagnosis. A clinical Magnetic Resonance Imaging (MRI) (methods described in the MRI Method section) was performed in the clinical confirmation phase to test for the presence of exclusion criteria and to assess the degree of brain damage at T0.

Patients diagnosed as MCI underwent a psycho-behavioural evaluation which included Alzheimer’s Disease Assessment Scale-cognitive (ADAS-Cog)^36^ to measure the severity of the most important symptoms of AD, and Neuropsychiatric Inventory (NPI)^37^, a scale administered to the informant, to evaluate severity and frequency of 12 neuropsychiatric disturbances common in dementia. The clinical MCI evaluation provided the baseline (T0) cognitive assessment.

Of the 131 MCI subjects, 1 died, 7 withdrew before randomization and 10 were excluded from the study for co-morbidities. Therefore, 113 MCI subjects have been randomized, resulting in 55 being assigned to the training group (MCI-training) and 58 to the no training group (MCI-no training). Of these 113 MCI subjects, 62 (55%) were single domain amnesic (aMCI), 28 (50.9%) in the MCI-training and 34 (58.6%) in the MCI-no training groups. The complete subdivision of MCI subjects, -training and –no training, in the different MCI subgroups is reported in Suppl. Table 1.

#### Randomisation

Subjects were randomly assigned to the multidomain training group (MCI-training) or to the no training group (MCI-no training) in a 1:1 ratio, considering a computer generated simple randomisation sequence. Randomisation was performed after the baseline assessment by a statistician not otherwise involved in the study and with no contact with the study participants. Outcome evaluators were and will be blinded to the allocation performed and participants were given instructions not to reveal their group assignment to the outcome evaluators. Clinicians and psychologists involved in the training were different from outcome evaluators. In case of failure in keeping outcome evaluators blinded to the allocation performed, different trained outcome evaluators were involved.

113 MCI subjects have been randomised, 55 being assigned to the training group (MCI-training) and 58 to the no training group (MCI-no training). Data were collected at the Department of Clinical and Experimental Medicine-Neurology Unit, University of Pisa & AOU Pisa, Institute of Clinical Physiology CNR, and Stella Maris IRCSS of Calambrone (PI).

### Outcomes

The primary outcome measure was the extent of cognitive decline from cognitive baseline (T0), assessed by ADAS-Cog at the end of 7 months of training/usual life (T7), and in a follow up re-evaluation performed 12 months after the end of training/usual care (T19). In this paper we report only the data for the cognitive changes between T0 and T7; the follow-up data will be reported in a subsequent paper.

Secondary outcome measures were: modifications, with respect to T0 and within task-related brain regions, in blood oxygen level dependent (BOLD) signal elicited by a cognitive task, as measured by functional Magnetic Resonance Imaging (fMRI) at T7 and T19; loss of grey and white matter in the cortex and the hippocampus with respect to T0, measured through Magnetic Resonance Imaging (MRI) at T7 and T19; modifications in cerebral blood flow with respect to T0, measured through MRI at T7 and T19. In this paper we report only the data for changes between T0 and T7; the follow-up data will be reported in a subsequent paper.

The general hypothesis was that the training would: i) reduce the cognitive decline; ii) reduce Gray Matter (GM) volume loss in hippocampus and parahippocampal areas; iii) modify Cerebral Blood Flow (CBF) in hippocampus and parahippocampal areas, as assessed by MRI, iv) modify task-related brain activity as assessed by fMRI.

### Intervention

The MCI-no training group continued with their usual life routine. The MCI-training group received a 7 month multidomain training, including cognitive, physical exercise and music therapy. At the end of 7 months of training/usual care (T7), both MCI-training and -no training subjects underwent a complete cognitive re-evaluation. A follow up re-evaluation will be performed 12 months after the end of training/usual care (T19).

No adverse event has been recorded in MCI-training and -no training subjects.

Train the Brain is a complex project including the screening, the intervention, the cardiovascular assessment, RM assessment, olfactometry, and animal studies. The total cost of the project has been 5 million Euros, 4 million of which provided by Fondazione Pisa. The cost of the sole intervention has been 400.000 Euros; in addition, the cost of building the new structure devoted to the project and fitting it with the necessary training equipment has been 500.000 Euros.

#### Cognitive training and stimulation of social interactions

Enrolled subjects were assigned to mixed-gender classes of 7–10 subjects each and given 2 sessions of supervised cognitive training of 60 min each per day, 3 times a week, in the morning, every other day from Monday to Friday (for a total of 6 hours per week), in the structure built on purpose within the National Research Council Area of Research in Pisa. The formation of small groups was intended to favour effective supervised training and to promote social interactions. Interestingly, supervised training in small groups resulted as the most effective protocol in the recent meta-analysis of cognitive training in MCI subjects by Gates and Sachdev^38^. The choice to explicitly insert in the training program activities which provided social stimulation stemmed from evidence in the literature showing the importance of social components as protective factors against major cognitive decline^8^,^39^.

The cognitive training programme was based on 8 cycles; each cycle was composed of 18 sessions of cognitive stimulation, with exercises and activities aimed at stimulating multiple cognitive functions. Each cycle lasted 3 weeks, after which the same kind of cognitive stimulation sessions were re-started, with exercises and activities of increased complexity compared to the previous cycle. Each cycle was structured in sessions aimed at stimulating the following main functions: acoustic attention, visual attention, visual memory, imagination, orientation and spatial memory, personal and temporal orientation, verbal memory, lexical abilities, memory for terms and meanings, affective memory, memory for texts, memory for faces and names, logic. Each cycle began with a theoretic-strategic lesson given by the cognitive trainers and focused on specific cognitive processes, such as memory, learning, attention, meta-cognition.

During the various sessions, there was an alternation of paper and pencil tests, social games, and multimedia-computer exercises, and the cognitive training was based on a balanced alternation of sessions for single cognitive modality and sessions focused on multimodal activities. There was no overlap between any paper and pen test and any of the neuropsychological tasks employed for cognitive assessment. All paper and pen exercises have been purposefully designed to train cognitive functions employing tasks never included in any of the cognitive tests.

Single modality sessions were aimed at intensively stimulating specific cognitive domains, such as memory, attention and executive functions. Multimodal activities were aimed at reconstructing a more ecological environment for the enrolled subjects, in order to favour socialisation and interpersonal exchanges in an enriching context. Alternation between the two types of sessions allowed maintaining an active participation in the enrolled subjects, stimulating their curiosity and attention and reducing levels of anxiety and potentially negative stress factors. Moreover, an alternation between sessions for single cognitive modality and multimodal activities was also aimed at avoiding habituation processes and at favouring diffuse and generalised brain stimulation, in order to maximise the chance to positively impact on the subject everyday life.

Once a month, a session of movie watching and discussion (Cineforum) was given to all classes together.

Once a week, subjects of each class attended together a session of 1 h music therapy, during which they were both involved in listening activities and also directly engaged in singing, musical instrument playing and rhythmic movements.

#### Physical training

Supervised 7-month aerobic exercise training was performed in a Fitness Centre within the structure built on purpose within the National Research Council Area of Research in Pisa. The participants attended 1-hour lessons 3 times per week in small groups with a maximum number of 10. Each lesson was planned according to the American College of Sports Medicine guidelines (American College of Sports Medicine, Thompson WR, Gordon NF, Pescatello LS: ASCM’s guidelines for exercise testing and prescription. 8th edition. Philadelphia: Lippincott Williams & Wilkins; 2010) and included aerobic exercise on an ergometer cycle, whose duration was increased gradually from 10 to 20 minutes as the participants progressed through the exercise intervention, followed by exercises targeted to improve muscle strength, physical function (static and dynamic), neuromuscular control and flexibility.

### Magnetic Resonance Imaging (MRI)

MRI data were acquired using a GE HDxt 1.5 T Signa Neuro-optimized System (General Electric Healthcare) fitted with 40 mT/m and 120 mT/m/ms slew rate high-speed gradients.

The MR protocol included the following sequences: axial T2-weighted FSE (TR/TE = 3000/102 ms, NEX = 2, scan time = 2′36″); sagittal T2-weighted FSE (TR/TE = 6420/125 ms, NEX = 2, scan time = 4′50″); axial FLAIR (TR/TI = 10000/2500 ms, NEX = 1, scan time = 3′30″); axial T2*-weighted GRE (TR/TE = 1000/30 ms, NEX = 2, scan time = 4′ 52″); T1-weighted 3D FSPGR (TR/TE = 12650/5300 ms, prep time = 700 ms, NEX = 1, isotropic voxel = 1 × 1 × 1 mm^3^, scan time = 10′ 08″); 3D Arterial Spin Labeling (ASL) (TR/TE = 4850/10 ms, NEX = 4, pld = 2025 ms, scan time = 6′ 10″); GRE-EPI (TR/TE = 2500/50 ms, FA = 90°, isotropic voxel = 3 × 3 × 3 mm^3^, 33 interleaved axial slices without gap resulting in a partial ~10 cm brain coverage) for task related fMRI. MRI examination was performed at T0 and repeated at T7. Written informed consent for the MR exam was obtained from the subjects.

#### MRI Clinical score

To obtain the clinical MRI scores, employed to assess the degree of brain damage at T0, we analyzed the white matter alterations for each subject, using the White Matter Changes (WMC) based on the visual scale assessed by Wahlund *et al*.^40^, and the temporal atrophy, assessed as in Scheltens *et al*.^41^ by means of the so-called MTA scale (medial temporal lobe atrophy visual rating scale); after randomization, no difference in WMC or MTA scores was detected between MCI-training and –no training groups (see online additional Methods).

#### Volumetric and Perfusion MRI analysis

Prior to undergo multi-modality statistical analysis, MRI maps were registered to a study-specific template obtained by means of the DARTEL algorithm (Diffeomorphic Anatomical Registration Through Exponentiated Lie algebra)^42^. Such procedure ensures an accurate registration of the cortical and subcortical regions^43^, allowing for an inter-subject comparison of analogous cerebral structures (see on line additional methods).

Voxel-wise parametric statistical maps were generated by entering the co-registered structural (GM) and cerebral perfusion (CBF) datasets into a voxel-wise paired t-test analysis to evaluate group differences between T0 and T7 for both the MCI-training and –no training groups.

In the ROI-based analysis, for each subject the GM volumes and CBF values of the hippocampus and parahippocampal gyrus regions were extracted by using preselected probability ROIs extracted from the LONI Probabilistic Brain Atlas^44^ database [ http://loni.usc.edu] and subsequently registered to the MNI atlas.

Image pre-processing and voxel-wise analyses were performed using the SPM8 software package (Wellcome Department of Imaging Neuroscience, London, UK). ROI analysis was performed using the FSL software package^45^. Cerebral perfusion map generation and statistical analysis on ROI were performed using specific home-made scripts written in Matlab programming language (The MathWorks, Inc.).

### Functional MRI analysis

#### Participants

A subset of 63 MCI patients (25 MCI-No Training: 12 males, mean age: 74.2 ± 4.6 years; 38 MCI-Training: 19 males, mean age: 74.5 ± 5.2 years) underwent the functional magnetic resonance imaging experiment. During the brain scan, the subjects were asked to perform a visuo-spatial attention task previously validated in healthy adults and professional athletes^46^. Of note, 50 MCI subjects accomplished both the T0 and T7 acquisitions: 19 participants were part of the MCI–No Training group (9 males; mean age ± standard deviation: 74.6 ± 4.0), whilst 31 belonged to the MCI-training group (15 males; mean age ± standard deviation: 74.2 ± 4.7). There were no differences for age (t_(1,48)_ = 0.321; p-value = 0.749) or gender (χ2_(1)_ = 0.005; p-value = 0.822) between groups.

#### Stimuli and Experimental Paradigm

Participants were equipped with MR-compatible goggles (VisuaStim, Resonance Technology Inc.) that subtended ~30 horizontal and ~22.5 vertical visual degrees (deg) and with two response buttons, one held in the right and the other in the left hand. Both goggles and response pads were connected to a workstation running MATLAB Release 2010b 64-bit (The MathWorks Inc., Natick, MA, USA) and Psychtoolbox v3.0.9^47^, which were used to design and administer the stimuli as well as to record subject responses.

During each fMRI session, subjects were asked to perform a visuo-spatial attention task: covertly track four stimuli (i.e., two red dots and two blue dots) and press a response button whenever one stimulus hit the target of the same colour (see on line additional method section). This task was found to elicit an ample pattern of brain activity in healthy adults, mainly involving the motion-related extrastriate visual cortex (MT), the attentional fronto-parietal network, the premotor and supplementary motor cortex, as well as basal ganglia and thalamus^46^ (see on line additional method section).

#### Behavioral Data Analysis

For each subject and session, overall task accuracy was computed across the three functional runs as the sum of true positive – ‘hit’ (e.g., subject pressed with the right hand when the red dot hit the red target) and true negative responses – ‘correct rejection’ (e.g., subject did not press with the left hand when a red stimulus crossed the blue target), divided by the sum of true positive, true negative, false positive – ‘false alarm’ (e.g., subject pressed with the right hand when the blue dot hit the red target) and false negative responses – ‘miss’ (e.g., subject did not press with the left hand when a blue stimulus crossed the blue target).

#### fMRI Data Analysis

For each session and subject functional brain images were pre-processed and analyzed using AFNI^48^ and FSL^45^. After the DICOM images have been reconstructed and transformed into NIFTI format, a slice timing correction was performed by using the Fourier transform method (*3dTshift*). We then corrected for subject head movements during acquisition (*3dvolreg*) by using a rigid-body transformation to spatially align each brain volume to the last one of the third functional run (i.e., the closest in time to the high-resolution anatomical scan); the computed six motion parameters (x, y and z rotation and translation for each volume) have been also included in the general linear model during the activation estimation as confound variables. A Gaussian smoothing filter with a full-width at half maximum of 6 mm was applied (*3dmerge*) to improve signal-to-noise ratio and account for inter-subjects anatomical variability at group level. Next, the hemodynamic activity of each voxel was normalized across time (mean = 100) so that task-related BOLD signal was expressed as percent signal change. The three functional runs were then concatenated and for each voxel, a general linear model (*3dDeconvolve*) was carried out to determine brain regions that were engaged (i.e., activated) during task execution at single subject level. Afterwards, the obtained statistical maps were transformed into the MNI152 standard space by means of an affine registration (i.e., FSL-FLIRT; 12 degrees of freedom) to allow for group-level analyses. In order to assess the effects of the cognitive and physical training program on brain activity, fMRI analyses were restricted to brain regions involved in visuo-spatial attention at group level. In this regard, we computed a one-sample t-test (qFDR corrected < 0.005) including T0 acquisitions of MCI-No Training and -Training subjects pooled together. Thus, we obtained an average map of task-related activation at baseline (i.e., T0), as well as a set of regions of interest (ROIs) to test the effect of training program on task-related BOLD signal.

### Statistics

A previous study^49^ reported a mean ADAS-Cog score among MCI subjects of 9.92 ± 4.81. With 5% significance level and power 90%, a final sample size of 42 subjects per group was calculated to detect a difference of 3.5 points in ADAS-Cog score decline between groups. Assuming a drop out of 30%, 60 subjects per groups was calculated as sample size.

Characteristics at the baseline of the study (T0) between MCI-training and MCI-no training groups have been compared considering the χ^2^ test or Fisher’s exact method, for categorical variables. Quantitative variables were evaluated using Generalized Linear Models (GLM), after testing for homoschedasticity (Levene’s test; in case of heteroschedasticity the Welch’s ANOVA was considered) or the nonparametric Wilcoxon rank sum test. Shapiro-Wilk test and D’Agostino-Pearson test^50^ were considered to test the normal distribution of quantitative variables.

The distribution of baseline variables of subjects who completed the study and those of subjects who were lost to follow-up (T7) were compared to test the assumption that data were missing due to random factors. The missing data pattern was examined to detect possible patterns in the distribution of missing values. Mixed-effects models (Proc Mixed) were used to study changes in cognitive scores related to group (MCI-training; MCI-no training), time, and group × time interaction, adjusting for baseline cognitive score. Compound symmetry covariance structure and Tukey adjustment for multiple comparisons were considered. Effect sizes (ES) were calculated to determine the magnitude of the effects^51^. Intent-to-treat analyses were made using multiple imputation (Markov chain Monte Carlo multiple imputation) of missing outcomes, through Proc MI; 30 imputed datasets were then combined considering Proc MI Analyze.

Statistical significance was assumed for a p-value < 0.05. The analyses were performed using Sigma Stat 7.0 software and SAS statistical package, release 9.4 (SAS Institute Inc., Cary, NC).

#### MRI volume and CBF values

To determine whether there was a difference in the GM volumes and the CBF values of the hippocampus and the parahippocampal gyrus regions between the acquisitions at T0 and T7 for both the MCI-training and MCI-no training groups, a two-way Mixed-model ANOVA (within-subjects factor: time; between-subjects factor: treatment) and the appropriate post hoc test was performed. Significance level p < 0.05.

#### fMRI, performance in the behavioural task

We used a Linear Mixed Model analysis (SPSS v21) to establish whether task accuracy was significantly influenced by the training program (n = 63). ‘Treatment’ (MCI-No Training versus MCI-Training), ‘Time’ (T0 versus T7) and their interaction ‘Treatment x Time’ were included in the model as factors of interest, adding ‘Age’ as nuisance variable. Afterwards, post-hoc testing and proper multiple comparisons correction (p < 0.05 Bonferroni-Holm) were carried out to assess significance of: ‘Time’ effect for MCI-no training subjects (MCI-no training T0 versus MCI-no training T7), and for MCI-training group (MCI-training T0 versus MCI-training T7), ‘Treatment’ effect at T0 (MCI-no training T0 versus MCI-training T0) as well as at T7 (MCI-no training T7 versus MCI-training T7).

#### fMRI, BOLD signal

To test the effect of training program on task-related BOLD signal, we ran a Linear Mixed Model analysis (*3dLME*)^52^ within brain regions previously identified as significantly activated. ‘Group, time and their interaction group were included in the model as fixed factors. ‘Task accuracy’ and its evolution in time (i.e., both the intercept and slope) were considered as within-subject covariates (i.e., random factor) while ‘Age’ was introduced as between subjects confounding variable. We then applied post-hoc analysis within brain regions that showed a significant effect (p < 0.05 cluster corrected) for one of the main factors or their interaction, similarly to what has been done for the behavioral data analysis. Post-hoc comparisons were adjusted for multiple comparisons using Bonferroni-Holm tests.

## Results

### Subjects

A total of 393 subjects have been subjected to the screening and, among them, 288 went on to the phase of clinical confirmation (Fig. 1). Of the 288 subjects entered in the phase of clinical confirmation, 157 did not receive an MCI diagnosis (136 had a normal cognitive profile and 21 resulted sufficiently impaired, cognitively and functionally, to meet National Institute of Neurological and Communicative Disorders and Stroke/Alzheimer’s Disease and Related Disorders Association criteria for mild AD) and 131 received an MCI diagnosis. Before randomization, 1 subject died, 7 withdrew and 10 were excluded from the study for co-morbidities. 113 have been enrolled and randomized to the intervention (MCI-training, n = 55) or the control (MCI-no training, n = 58) group. Fifty three MCI-training and 50 MCI-no training subjects completed the training and the T0 and T7 cognitive assessments. There was a significant difference in the drop out rate between MCI-training and –no training groups (n = 2 (3.6%) and n = 8 (13.8%) respectively), with the latter exhibiting the higher drop-out rate (p = 0.011). Main reason for drop-out in MCI-no training was motivation.

After randomization, MCI-training and -no training groups resulted similar for age, education, screening- test scores and gender proportion (Table 1); in particular, mean age resulted around 75 years and mean MMSE score was slightly above 25 for both groups. The distributions of baseline variables of subjects who completed the study and of those who were lost to follow-up were not significantly different (data not shown).

### Effects of training on cognitive status and single cognitive domains

**Figure 2:**
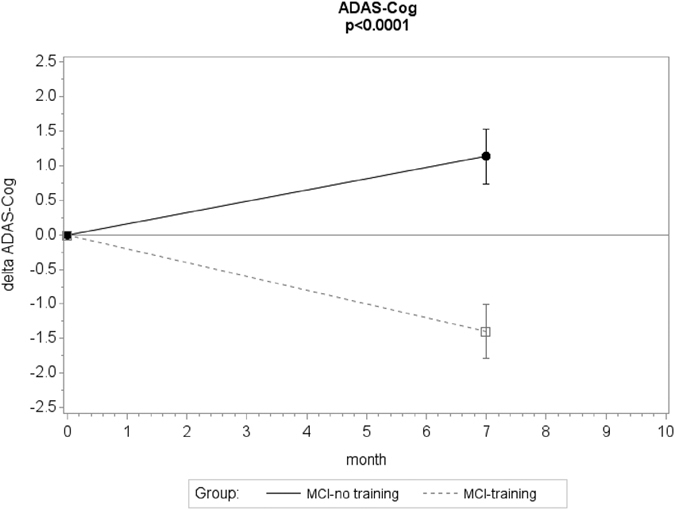
Changes in cognitive score at the ADAS-score during the 7-months intervention . Mean change in ADAS-Cog score from baseline (negative differences correspond to lower scores at T7 than T0, indicating performance improvement). Error bars are s.e.m.; p value from mixed model repeated measure analysis, group × time interaction.

A significant beneficial effect of the combined training on the primary outcome (ADAS-Cog) was detected (Fig. 2): the mean estimated change in ADAS-Cog at the 7-months follow-up was −1.40 (standard error (SE) = 0.32, p value 0.0007) in the MCI-training group and 1.15 (SE = 0.25, p value 0.026) in the MCI-no training group. The mean difference between groups in relation to change in ADAS-Cog was −2.17 (SE = 0.42; 95% CI (−2.99, −1.34)), statistically significant (p < 0.0001 for the interaction group × time). The correspondent effect size (ES) was in the medium range (ES = −0.55; 95% CI (−0.60, −0.50)).

25 MCI-training subjects (47%) showed a decrease in ADAS-Cog score higher than 1.5, and only 5 MCI-training subjects (9%) showed an increase in ADAS-Cog higher than 1.5; on the contrary, 20 MCI-no training subjects (40%) showed an increase in ADAS-Cog score higher than 1.5 and only 7 (14%) showed a decrease higher than 1.5 (differences between the two groups significant, z-test p < 0.001 in both comparisons).

**Figure 3:**
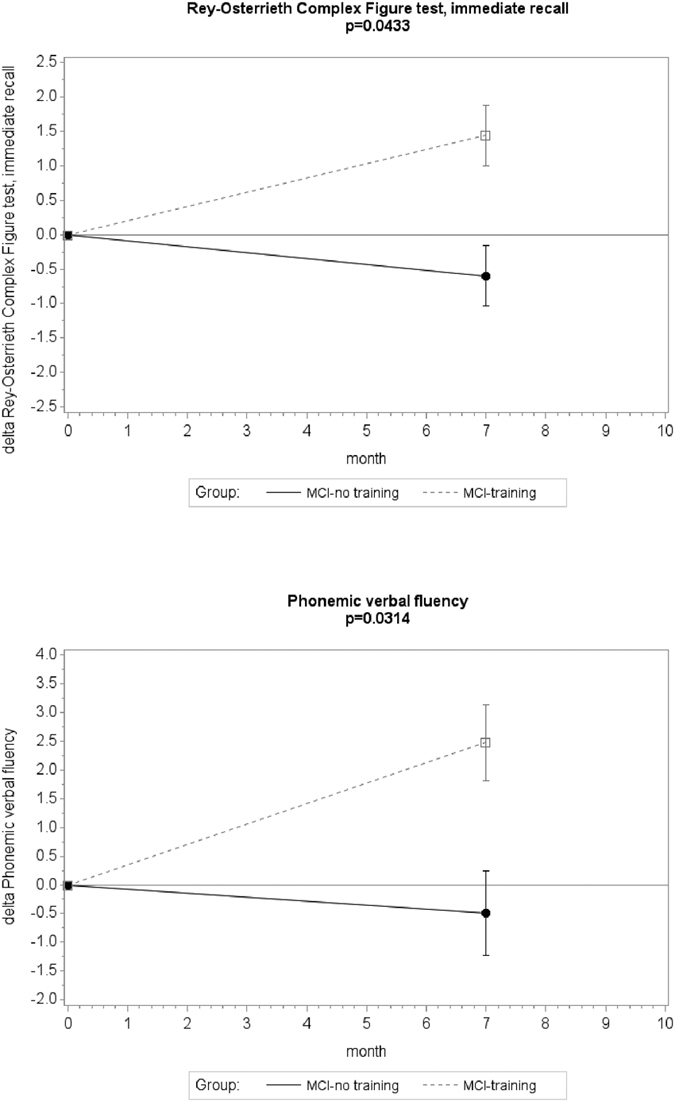
Estimated mean changes from baseline (T0) to T7 in cognitive score at the Rey-Osterrieth Complex Figure test immediate recall and Phonemic verbal fluency. Positive differences correspond to higher scores at T7 than T0, indicating performance improvement. Error bars are s.e.m.; p value from mixed model repeated measure analysis (group × time interaction).

Significant intervention effects were also observed in relation to the Rey-Osterrieth Complex Figure test immediate recall and the Phonemic verbal fluency, with, however, a small to medium effect size (0.37 and 0.32, respectively) (Fig. 3, Suppl. Table 2).

### Effects of training on Gray Matter loss and on Cerebral Blood Flow in the Medial Temporal Lobe (MTL)

Hippocampus and related structures of MTL are early targets of both physiological and pathological aging^53^. While greater amounts of physical activity have been associated with increased prefrontal and hippocampal volume or blood flow in healthy adults^13^,^54^, nothing is known for MCI subjects.

Effects of training on Gray Matter loss and on Cerebral Blood flow in the hippocampus and cortical structures of MTL was one of our secondary outcomes. The degree of atrophy and the change of perfusion between T0 and T7 in the hippocampus and parahippocampal areas were assessed in MCI-training and -no training subjects by means of 3D structural MR, evaluating separately gray matter and white matter volumes, and by ASL technique that allows the quantitative measurement of CBF.

In the volumetric and perfusion MRI assessment, we experienced a strong drop-out, unevenly distributed between the training and no training groups, since only 48 MCI-training and 22 MCI-no training subjects completed the T0 and T7 MRI sessions. We performed an analysis of the baseline characteristics reported in Table 1, age, education, screening- test scores and gender proportion, in these reduced groups of MCI-training and -no training. The results indicate that the MCI-training and -no training groups which underwent MRI did not differ for any of these variables between themselves and did not differ from the respective total cohort (Table 2 and Suppl. Table 3).

We found the same degree of hippocampal volume reduction between T0 and T7 in MCI training (n = 48, −0.6% ± 0.2) and –no training (n = 22, −0.64% ± 0.5) groups, with no significant time x treatment interaction (Two way mixed model ANOVA, factor time p < 0.01, p interaction > 0.05). No significant volume reduction was found in the parahippocampal region in either group.

**Figure 4:**
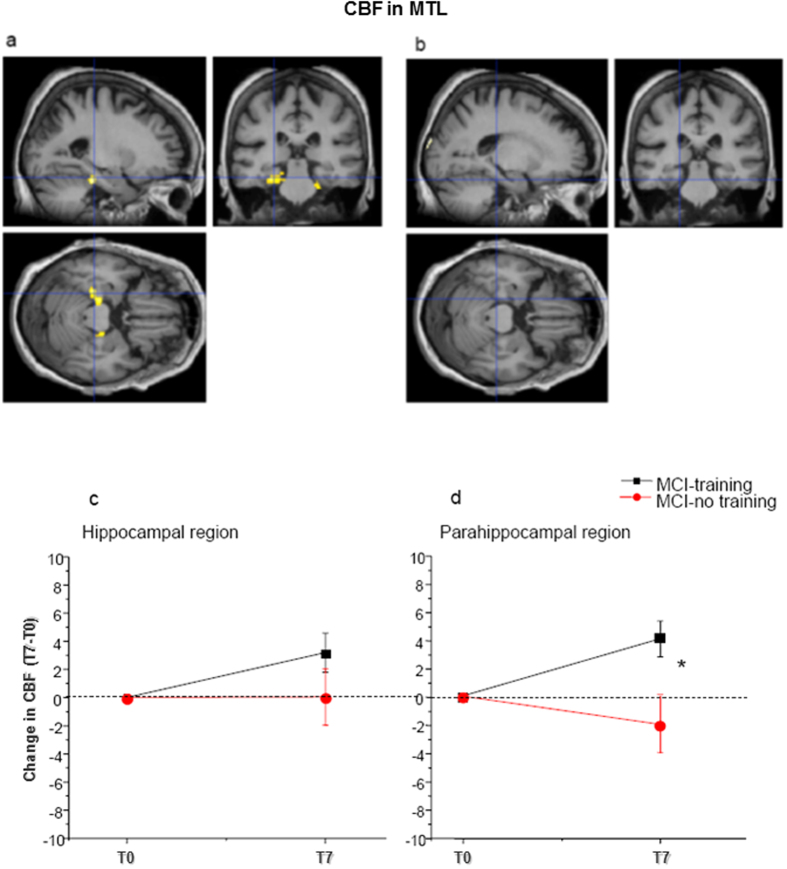
Train the Brain intervention increases Cerebral Blood Flow (CBF) in Medial Temporal Lobe (MTL) regions. Voxel-wise paired t-test analysis between T0 and T7 for both the MCI-training and –no training groups (**a**) Increase of CBF in the MCI-training group (n = 48) at T7 with respect to T0 (p threshold 0.001). (**b**) No significant change of CBF between T0 and T7 was found in the MCI-no training group (n = 22). Lower line/row). Change of CBF during the 7 months intervention in (**c**) the hippocampus and (**d**) the parahippocampal regions Mean change from baseline is presented (positive differences correspond to higher CBF at T7 than T0. Error bars are s.e.m. In the parahippocampal region, two way mixed model ANOVA, time x treatment, revealed a significant time x treatment interaction (p < 0.05) with MCI-training subjects showing a significant improvement (*) and MCI-no training subjects showing a not significant decrease.

CBF was increased in the hippocampal and parahippocampal regions of MCI-training subjects (n = 48) between T0 and T7 (+3.2 ± 1.4% for the hippocampus and + 4.1 ± 1.2% for the parahippocampus), but statistical significance was reached only for parahippocampal regions; no significant change was found in MCI-no training subjects (n = 22, CBF difference T7-T0 + 0.043 ± 2% in the hippocampus and −1.8 ± 2% in the parahippocampus) (Two way mixed model ANOVA, p interaction time x treatment p > 0.05 for the hippocampus and < 0.01 for the parahippocampus, post-hoc analysis for parahippocampus, Dunn-Sidak method, MCI-training subjects T0 vs T7 p < 0.05, MCI-no training subjects, T0 vs T7 p > 0.05) (Fig. 4, Table 3).

### Effects of training on task-related brain activity

Modification in brain activity elicited by a cognitive task was one of our secondary outcomes. Our hypothesis was that the training program would modify the BOLD response within task-related brain regions. In particular, it has been suggested that neural efficiency is inversely proportional to the recruitment of brain networks during cognitive tasks: for a given performance level, subjects more skilled and more efficient in dealing with cognitive load would show less brain activation^46^,^55^.

Thus, in a subset of subjects (n = 63 MCI patients: n = 25 MCI-No Training, 12 males, mean age 74.2 ± 4.6 years; 38 MCI-Training, 19 males, mean age 74.5 ± 5.2 years), we tested whether our training program would modify the pattern of brain activity elicited by a visuo-spatial attention task, previously validated in a population of adult subjects^46^. There were no differences for age (t_(1,48)_ = 0.321; p = 0.749) or gender (χ^2^_(1)_ = 0.005; p = 0.822) between these MCI-training and –no training groups.

### fMRI, performance in the behavioural task

**Figure 5:**
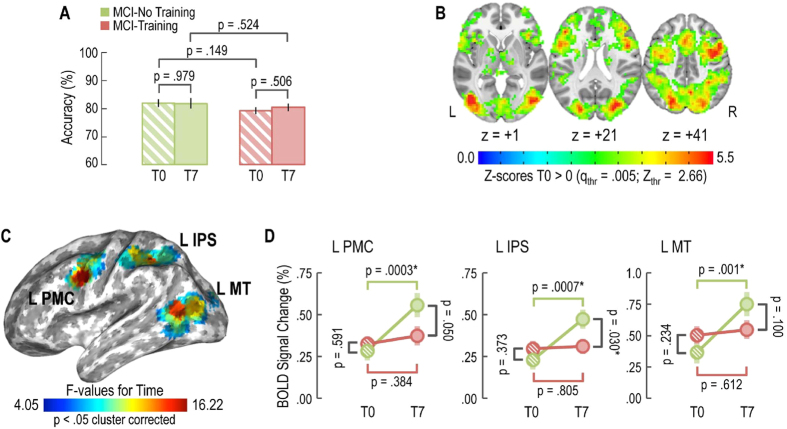
Training maintains neural efficiency as measured by task-related fMRI. (**A**) No effect of training on task accuracy was evident for MCI-training and -no training subjects. (**B**) T0 task-related regions of interest obtained by pooling MCI-training and -no training subjects together. (**C**) Effects of training on BOLD signal: brain regions showing a significant effect for the ‘Time’ factor (L MT: left middle temporal motion-related region; L IPS: left intraparietal sulcus; L PMC: left premotor cortex). (**D**) Post-hoc analysis for each of the regions in section C, MCI-no training subjects showed an increase of BOLD signal between T0 and T7, while MCI-no training subjects did not, suggesting that our training program maintains neural efficiency.

Results for the Linear Mixed Model (Fig. 5A) showed no effect for ‘Treatment’ (p = 0.157), ‘Time’ (p = 0.694), or ‘Treatment x Time’ interaction (p = 0.664), indicating that task accuracy was not affected by training program. Specifically, MCI-No Training subjects demonstrated (mean ± standard error) 81.9 ± 1.4% [95 CI: 79.0–84.8] task accuracy at T0 and 81.8 ± 1.8% [95% CI: 78.3–85.4] task accuracy at T7. On the other hand, MCI-Training patients achieved 79.2 ± 1.2% [95% CI: 76.9–81.5] task accuracy at T0 and 80.4 ± 1.4% [95% CI: 77.6–83.2] at T7.

### fMRI Data

The baseline (i.e., T0) task-related brain response obtained by pooling MCI-training and -no training subjects together (q FDR = 0.005; Fig. 5B) entailed a wide bilateral set of brain regions: the primary visual cortex, the posterior portions of the middle temporal gyrus involved in motion processing (MT), the occipito-parietal components of the dorsal stream and attention network (middle and superior occipital gyrus, intraparietal sulcus and superior parietal lobule) as well as frontal lobe regions that are related to action planning, motor control and focusing (ventral and dorsal premotor, supplementary motor area).

Within these ROIs, the Linear Mixed Model highlighted three brain regions of the left hemisphere that showed a significant effect for the ‘Time’ factor (p < 0.05 cluster corrected 2214 mm^3^; Fig. 5C): L MT (MNI coordinates: x = −39, y = −72, z = 12;), L IPS (MNI coordinates: x = −39, y = −45, z = 39;) and L PMC (MNI coordinates: x = −51, y = 0, z = 30;). For each of these three regions, post-hoc analyses (Fig. 5D) revealed a time-dependent increase of BOLD activity for the MCI-no training group (L MT: F_(1,48)_ = 11.909; p-value = 0.001; L IPS: F_(1,48)_ = 13.016; p-value = 0.0007; L PMC: F_(1,48)_ = 15.357; p-value = 0.0003), but not for the MCI-training group (L MT: F_(1,48)_ = 0.260; p-value = 0.612; L IPS: F_(1,48)_ = 0.062; p-value = 0.805; L PMC: F_(1,48)_ = 0.771; p-value = 0.384). Thus, only MCI-no training subjects show an increase of BOLD signal between T0 and T7, suggesting that neural efficiency is diminished in these subjects, while it is preserved in MCI-training subjects.

## Discussion

To our knowledge, this is the first randomized control trial to investigate in MCI elders the impact of the combination of physical and cognitive training not only on cognitive decline (as in ref. 56) but also on markers of brain damage. The training was relatively intensive, three mornings each week, and all activities were supervised. Epidemiological studies suggested the possibility that unsupervised, subject-choice physical or cognitive activities might also be beneficial in reducing age related cognitive decline. However, since no study was available, when the study was undertaken, directly investigating the effects of a combined physical and cognitive training in MCI subjects, we felt the need to provide supervised, reproducible activities. In addition, the presence of psychological supervision contributed to the high compliance for the training exhibited by our subjects. The fact that training activities were inspired by everyday activities made possible for each participant to find matching daily-activities. The choice of a supervised, small group training received *a-posteriori* support by the review of Gates and Sachdev^38^ on the effects of cognitive training in preclinical and early AD and commenting that supervised small group multi-domain training providing the greatest benefits.

The intervention was safe and well received, as shown by the very low drop out rate in the MCI-training group. This high compliance underscores the advantage of a multi component training in terms of motivation; indeed our intervention not only combined physical and cognitive training but provided a variety of activities, included music therapy, aiming to stimulate social interactions and to reduce adaptation and decline of interest. The social component is often neglected in interventions, but being engaged in social activities resulted a protective factor in the Yaffe *et al*.^8^ study and there is evidence that mental, physical and social stimulation equally contribute to decrease dementia risk^39^. Thus, including social components in future interventions might prove advantageous.

Our primary outcome was the extent of cognitive decline from cognitive baseline and the end of 7 months of training/usual life, with the general hypothesis that the training would reduce the extent of cognitive decline with respect to controls. Our results show an effect of the training stronger than hypothesized: indeed, 7 months of training significantly improved global cognitive status, as assessed with ADAS-Cog score, while control subjects show a significant decline during the same period. Thus, it appears that the intervention does not simply restrain cognitive decline, but causes an improvement with respect to baseline cognitive status. The effect size for the improvement is at the upper limit of the small effect size range; it is therefore bigger than effect size found in either physical only or cognitive only training interventions in MCI subjects^21^,^38^. Compared with the only other combined physical and cognitive intervention performed in an MCI population^56^, the effect size of the improvement in training subjects is similar; however, the cumulative effect size is larger in our study, since performance of our control MCI subjects significantly declined over the training period, while in (56) it did not. This could be due to the fact that in ref. 56 the control group received both sham cognitive and sham exercise interventions, such as watching 5 short National Geographic videos followed by a set of 15 questions regarding the presented material, while our MCI-no training group continued with their usual life.

Our results are also in line with the results obtained in a large population of cognitively unimpaired elders by the FINGER study^17^, a multicomponent intervention administered for two years. In this study both the intervention and non intervention groups showed an improvement in cognitive performance, with the intervention group showing a significantly larger improvement in global cognition than the control group. Here it is more difficult to compare effect size of interventions, since our population of subjects is MCI and the population of FINGER is not. In addition, the FINGER control group received regular health advice on healthy diet and physical, cognitive, and social activities beneficial for management of vascular risk factors and disability prevention, which our MCI control group did not receive.

Even taking into account differences in intervention protocols, these studies and our study suggest that combined physical and cognitive interventions might indeed be particularly effective in improving cognitive performance both in non MCI and in MCI subjects.

Cognitive improvement was evident also at the level of single cognitive domains and in particular for phonemic verbal fluency. Thus, the Train the Brain combined intervention has beneficial effects both on global cognition and on cognitive domains of relevance for everyday activities.

Concerning the secondary outcomes, the general hypothesis was that the training would: reduce the Gray Matter (GM) volume loss in hippocampus and parahippocampal areas, modify Cerebral Blood Flow (CBF) in hippocampus and parahippocampal areas, as assessed by MRI, and modify task-related brain activity as assessed by fMRI.

Gray Matter volume loss turned out to be the same in MCI-training and MCI-no training subjects, indicating that our training had no effect on this outcome. Therefore, the beneficial effects of training on cognitive status cannot be attributed to an effect on hippocampal atrophy, which was the same in MCI-training and –no training subjects. This is reminiscent of data from animal models of neurodegenerative disorders^15^,^57^ which show that increasing physical and cognitive activity can ameliorate cognitive performance without reducing brain atrophy, acting instead on factors involved in brain functional plasticity. In the human literature, hippocampal volumetry has been connected with complex cognitive activity in aging by Valenzuela *et al*.^58^, who determined whether individual differences in lifespan complex mental activity are linked to altered rates of hippocampal atrophy. They found that high level of complex mental activity across the lifespan was correlated with a reduced rate of hippocampal atrophy. However, in a more recent study on post mortem samples from subjects with a 14 years follow up, coming from a large cohort of subjects aged > 65 years (United Kingdom Medical Research Council Cognitive Function and Ageing Study), Valenzuela *et al*.^59^ found no evidence for hippocampal neuroprotection in subjects with high levels of complex cognitive activity. Instead, men and women both exhibited significantly greater neuronal density, as well as correlated increases in cortical thickness in prefrontal area 9 linked to cognitive lifestyle, consistent with a compensatory process. Lifespan complex cognitive activity may therefore protect against dementia through multiple biological pathways.

As for physical exercise effects on hippocampal volumetry, data are available for non MCI elders: Erickson *et al*.^60^, in a randomized controlled trial in cognitively normal older adults, showed that aerobic exercise training leads to improvements in spatial memory and is accompanied by a 2% increase in anterior hippocampal volume, which reverses age-related volume loss. They also demonstrated that increased hippocampal volume is associated with greater serum levels of BDNF. BDNF is a mediator of neurogenesis in the hippocampal dentate gyrus in response to physical exercise and EE but also a well know synaptic plasticity factor (see ref. 15).

As for the other two secondary outcomes, data of MRI and fMRI show that beneficial effects of intervention were evident on brain CBF and neural processing efficiency, suggesting an improvement of cerebral function and, possibly, a direct action on dementia pathogenesis. Transition from healthy aging to MCI to AD seems to be accompanied by a complex pattern of changes in the pattern of CBF (see ref. 61). Arterial Spin Labelling MR offers a non invasive method to assess Cerebral Blood Flow and is an emerging biomarker for MCI and AD^62^. Diminished CBF reduces brain supply of oxygen, energy substrates and nutrients, impairs the clearance of neurotoxic molecules, and this is known to contribute to neuronal dysfunction found in neurodegenerative disorders, which are of early appearance in Medial Temporal Lobe regions. Our results show that MCI subjects respond to training with an increase in Medial Temporal Lobe CBF, particularly evident in parahippocampal regions, strongly involved in non verbal, spatial information processing (see ref. 63). Recently, it has been suggested that reduced CBF might directly contribute to brain beta-amyloidosis^64^. Indeed, in animal models, mild chronic brain hypoperfusion results in increased brain A-beta presence^65^. Therefore, the increase in CBF caused by the Train the Brain intervention might not only indicate a more physiological perfusion state, with a reduction of neuronal dysfunction, but might also affect brain A-beta burden.

An effect of lifestyle factors such as cognitive activity on Aβ is also suggested by human epidemiological studies. Landau *et al*.^66^ assessed whether the level of cognitive and physical exercise was associated with the extent of Aβ deposition in healthy elders; Aβ burden was determined using the 11–labeled Pittsburgh Compound B and positron emission tomography. The subjects were followed for 6 years. The results showed that lower Aβ burden was found in subjects more engaged in cognitively stimulating activities, particularly during early and middle life. A recent study suggests that greater levels of physical activity in MCI subjects is associated with lower Aβ plaque and tangle deposition, estimated by PET^67^. Thus, there seems to be a correlation between cognitive/physical activity and Aβ burden.

A recent finding in animal models of AD, which has been shown also in human patients, is that age-related cognitive impairments correlate with the presence of diffuse, low-weight Aβ oligomers, which are sufficient to impair synaptic plasticity and memory performance also when acutely administered to young normal animals (see ref. 68, 69, 70), and are currently hypothesized to be the first pathogenic agents in AD. It has been shown^71^ that early exposure to increased levels of physical and cognitive activity in social setting (Enriched Environment, EE) completely prevented Aβ soluble oligomers extracted from human AD patient cortex from damaging hippocampal synaptic plasticity in young normal animals. Thus, physical and cognitive activity could not only reduce Aβ in the aged brain, but also make age-vulnerable brain structures, such as the hippocampus, resilient to Aβ negative effects.

An indication of an effect of the combined physical and cognitive intervention on brain functional plasticity comes from the fMRI data, suggestive of an effect of training on neural efficiency. Indeed, while performance levels remained equal between T0 and T7 in both groups, MCI-no training subjects showed an increase of BOLD signal between T0 and T7, an indication of reduced neural efficiency; on the contrary, neural efficiency seems to be preserved in MCI-training subjects, who achieved the same behavioural performance accuracy engaging less neural resources.

A point of weakness of this study is the reduction we experienced in the sample of subjects undergoing MRI, and in particular the fact that this reduction was larger for MCI-no training than for MCI-training subjects. This may have reduced the benefits of the initial randomization and could weaken the conclusions which can be drawn from MRI data. However, the MCI-training and -no training groups which underwent MRI did not significantly differ for any of the baseline variables between themselves and did not differ from the respective total cohort, suggesting that in these smaller groups distribution of baseline data respects the one in the original, randomized groups. Nevertheless, the differential drop out remains a point of weakness and, in a future work, it would be necessary to program supervision also for the controls to maintain motivation, interest and high compliance for the entire duration of the study, reducing their drop out. We cannot exclude that, even if not significant, imbalance in an important parameter such as education between MCI-training and –no training group which underwent MRI might have contributed to an imbalance in MRI results. However, MRI results refer to a change following intervention, and therefore one should further hypothesize that cognitive reserve affect training effects. In a next work, it will be important to explicitly investigate the possible role played by education in terms of training effectiveness.

One final consideration on our data is that in this paper we report acute training effects on cognition, CBF and brain functional activation, assessed immediately after training end. Follow up data, which are underway, will tell us if and how these effects decay with time and will allow a better understanding of the clinical relevance of our results in terms of dementia onset delay.

In conclusion, a non pharmacological, combined physical and cognitive training in a social setting improves cognitive status of MCI subjects and improves indicators of brain health. The improvement is a small effect size. However, as pointed out by Ngandu *et al*.^17^, even small size effects in these initial stages may result in considerable gains in terms of public health. This underscores the importance of interventions aimed at multiple lifestyle factors as possible strategies to reduce or delay dementia conversion in MCI subjects, thus reducing dementia incidence.

## Additional Information

**How to cite this article**: Maffei, L. *et al*. Randomized trial on the effects of a combined physical/cognitive training in aged MCI subjects: the Train the Brain study. *Sci. Rep.* **7**, 39471; doi: 10.1038/srep39471 (2017).

**Publisher's note:** Springer Nature remains neutral with regard to jurisdictional claims in published maps and institutional affiliations.

## Supplementary Material

Supplementary Methods

## Figures and Tables

**Table 1:**
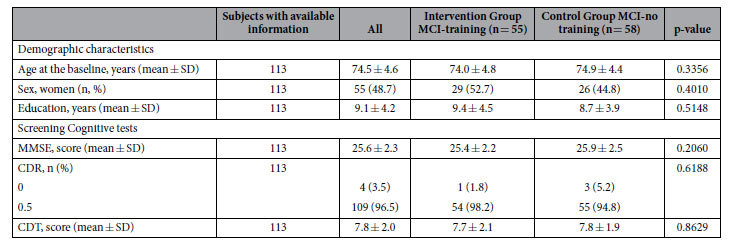
Baseline characteristics of subjects who were randomized to the trial (data are expressed as n, n (%), mean ± SD).

**Table 2:**
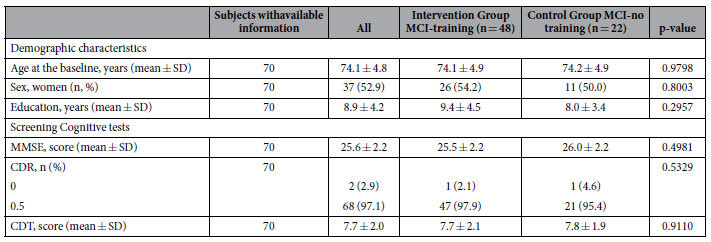
Baseline characteristics of subjects who were randomized to the trail and have available data for RM (data are expressed as n, n (%), mean ± SD).

**Table 3:** Training increases CBF in parahippocampal regions.

	MCI training T0	MCI training T7	MCI NO training T0	MCI NO training T7
CBF hipp.	63,2 ± 1,4	66,4 ± 1,2	66,5 ± 2,0	66,6 ± 1,6
CBF parahipp. gyrus	64,9 ± 1,2	69 ± 1,1*	68,6 ± 1,4	66,8 ± 2,0

Values of Cerebral Blood Flow (CBF) at T0 and T7 for MCI-training (n = 48) and –no training subjects (n = 22). Asterisks denote significant difference between T0 and T7. Two way RM ANOVA, interaction time × treatment p < 0.05 for parahippocampal gyrus.
