# Supplementary material for: Randomized trial on the effects of a combined physical/cognitive training in aged MCI subjects: the Train the Brain study
Source: Sci Rep. 2017 Jan 3;7:39471. doi: 10.1038/srep39471 (PMC5206718; doi:10.1038/srep39471)
Supplement: Supplementary Methods [file srep39471-s1.pdf]

Randomized trial on the effects of a combined physical/cognitive training in aged MCI subjects: the Train the Brain study.

Authors:
The "Train the Brain" consortium

Additional Methods section

Diagnostic criteria proposed by European Consortium on Alzheimer's Disease Working Group on MCI [1]:

(1) Cognitive complaints corroborated by an informant;

(2) The reporting of a decline in cognitive functioning relative to previous abilities during the past year by the patient or informant;

(3) Cognitive disorders as evidenced by clinical evaluation (impairment in memory or in another cognitive domain);

(4) no or minimal impairment in activities of daily living as determined by a clinical interview with the patient and informant (ADCS MCI-ADL Scale), a modification of the ADCS-ADL Scale designed to increase sensitivity to impairments in instrumental activities that may occur in MCI.

Neuropsychological battery

Short term memory was evaluated with digit span forward [2], Corsi test forward [2], Babcock short story (BSS, [3]) immediate recall (IR, immediate repetition after reading), Rey Auditory Verbal Learning Task (RAVLT, [4]) Immediate Recall (IR, immediate repetition after reading) and the Rey-Osterrieth Complex Figure Test (ROCF, [3]) immediate recall (IR, 1 minute after copy).

Retrospective memory was assessed using Babcock short story (BSS, [3]) delayed recall (DR, 20 minutes after after reading), Rey Auditory Verbal Learning Task (RAVLT, [4]) delayed recall (DR, 15 minutes after after reading) and the Rey-Osterrieth Complex Figure Test (ROCF, [3]), delayed recall (DR, 20 minutes after the copy).

Visuo-spatial abilities and non verbal intelligence were measured with the Raven Coloured Progressive Matrices (CPM-47, [4]).

Executive functions and attention were assessed using the phonemic verbal fluency (PVF, [4]), semantic verbal fluency (SVF, [5]), the Trail Making Test (TMT A-B) [6] and attentional matrices (AM, [7]).

Constructional praxis was measured with the copy of Rey-Osterrieth Complex Figure Test

and with the copy of freehand drawings (CFD, [4]) and the copy of drawings with programming elements (CFDP, [4]).

Protocol of the behavioural task used for fMRI

The task consisted of covertly tracking four stimuli (i.e., two red dots and two blue dots) while gazing a central fixation point. The four dots were moving randomly at a constant speed of 6 deg/s. Two near-peripheral (i.e., 7.5 deg of eccentricity) coloured targets were located to the right (red target) and left (blue target) of the fixation point. Participants were asked to press the correct button whenever one stimulus hit the target of the same colour (e.g., press the right hand button if one of the red dots crosses the red target) and their performance was recorded in real-time. For each functional run, three blocks of task, lasting 30 seconds each, were alternated with four resting intervals (15 seconds long), during which only the two static targets and the fixation point were visible on the screen. In order to familiarize the subjects with task procedure, prior the actual measurements they were provided a brief training session outside the scanner that endured until at least 66% accuracy was obtained. Usually two, and up to four task sessions were necessary so that every participant reached this threshold.

MRI clinical score

The White Matter Changes (WMC) is based on the visual scale assessed by Wahlund et al. [8] using the T2-weighted FSE acquisitions and FLAIR images: five different regions were rated (ranging from 0 to 3) in the right and left hemispheres separately: (1) the frontal area, which was the frontal lobe anterior to the central sulcus; (2) the parieto-occipital area, which consisted of the parietal and occipital lobes together; (3) the temporal area, which was the temporal lobe (the border between the parieto-occipital and temporal lobes was approximated as a line drawn from the posterior part of the Sylvian fissure to the trigone areas of the lateral ventricles); (4) the infratentorial area, which included the brain stem and cerebellum; and (5) the basal ganglia, which included the striatum, globus pallidus, thalamus, internal and external capsules, and insula.

After randomization, no difference in WMC scores between MCI-training and MCI-no training group was present (frontal region, median score 1, interquartiles [0,1] in both groups, Mann-Whitney Rank Sum Test, $p = 0.91851$; parieto occipital lobes median score 0 [0.1] for MCI-training, 1 [0.1] for MCI no training, Mann-Whitney Rank Sum Test, $p = 0.17$; temporal lobe, median score 0 [0.0] for both groups, Mann-Whitney Rank Sum Test, $p = 0.99$; infratentorial, median score 0 [0.0] for both groups Mann-Whitney Rank Sum Test, $p = 0.79$; basal ganglia median score 0 [0.0] for both groups, Mann-Whitney Rank Sum Test, $p = 0.72$).

Temporal atrophy was assessed as in Scheltens et al. [9] by means the so-called MTA scale (medial temporal lobe atrophy visual rating scale): the MTA is based on a visual score on the 3-planes T1 weighted images (3D high resolution FSPGR) of the height of the hippocampus and the width of the surrounding CSF space. The severity of medial temporal lobe atrophy (MTA) is scored from 0 (no atrophy) to 4 (most severe atrophy), to each side of the brain. After randomization, no difference in MTA scores between MCI-training and MCI-no training group was present (median score 2, interquartiles [1,2] in both groups, (Mann-Whitney Rank Sum Test, $p = 0.57993$).

DARTEL algorythm

MRI data pre-processing included the following steps: (1) checking for scanner artefacts for each subject; (2) setting the image origin to the anterior commissure; (3) SPM default segmentation of brain tissues for the extraction of the Grey Matter (GM) probability maps; (4) implementing the DARTEL procedure to generate a study-specific template; (5) computing the Cerebral Blood Flow (CBF) maps by means of the Arterial Spin Labeling (ASL) technique; (6) performing affine transformation of the GM maps and the CBF maps into the MNI space; (7) applying standard smoothing to the obtained maps (i.e. with 6-mm isotropic Gaussian kernel). After this pre-processing step, smoothed modulated normalized data (in the MNI space) were obtained for subsequent analysis.

References

1. F. Portet, P. J. Ousset, P. J. Visser et al., *J Neurol Neurosurg Psychiatry* **77** (6), 714 (2006); B. Winblad, K. Palmer, M. Kivipelto et al., *J Intern Med* **256** (3), 240 (2004).
2. A. Orsini, D. Grossi, E. Capitani et al., *Ital J Neurol Sci* **8** (6), 539 (1987).
3. G.A. Carlesimo, I. Buccione, and L. Fadda, *Nuova Rivista di Neurologia* **12**, 1 (2002).
4. G. A. Carlesimo, C. Caltagirone, and G. Gainotti, *Eur Neurol* **36** (6), 378 (1996).
5. G. Novelli, C. Papagno, E. Capitani et al., *Archivio di Psicologia. Neurologia e Psichiatria* **47**, 477 (1986).
6. A. R. Giovagnoli, M. Del Pesce, S. Mascheroni et al., *Ital J Neurol Sci* **17** (4), 305 (1996).
7. H. Spinnler and G. Tognoni, *The Italian Journal of Neurological Sciences* **Supplement 8** (1987).
8. L. O. Wahlund, F. Barkhof, F. Fazekas et al., *Stroke* **32** (6), 1318 (2001).
9. P. Scheltens, D. Leys, F. Barkhof et al., *J Neurol Neurosurg Psychiatry* **55** (10), 967 (1992).

Supplementary Table 1.

Composition of –training and –no training groups in terms of MCI subtypes.

	**Total subjects (n=113)**	**Intervention Group MCI-training (n=55)**	**Control Group MCI-no training (n=58)**
**Sub-types of MCI**			
aMCI	62	28 (50.9 %)	34 (58.6 %)
naMCI	16	4 (7.2 %)	12 (20.6 %)
aMCI multidomain	31	22 (40.0 %)	9 (15.5 %)
naMCI multidomain	4	1 (1.8 %)	3 (5.1 %)

Supplementary Table 2.

	Baseline, Post-training T7 score, within-group mean differences at T7								*Between-group mean differences at T7*		
	MCI-training group				MCI-no training group						
	Baseline Mean (SE)	Post-training T7 Mean (SE)	Mean Difference (SE)	p-value	Baseline Mean (SE)	Post-training T7 Mean (SE)	Mean Difference (SE)	p-value	Mean Difference (SE)	p-value[§]	Effect size d
Digit span forward	5.46 (0.10)	5.36 (0.09)	-0.10 (0.09)	0.3717	5.33 (0.08)	5.26 (0.08)	-0.04 (0.07)	0.5559	-0.01 (0.11)	0.8294	-0.04
Corsi test forward	4.58 (0.06)	4.49 (0.05)	-0.09 (0.07)	0.3093	4.58 (0.06)	4.52 (0.06)	-0.09 (0.07)	0.5242	0.03 (0.08)	0.7808	-0.05
Rey Auditory Verbal Learning Task immediate recall	29.63 (0.67)	31.70 (0.76)	1.97 (0.69)	0.0163	31.85 (0.54)	33.54 (0.76)	1.76 (0.51)	0.0483	0.12 (0.87)	0.7528	0.06
Babcock short story immediate recall	3.74 (0.16)	4.37 (0.19)	0.63 (0.20)	0.0444	4.54 (0.17)	4.58 (0.17)	0.08 (0.24)	0.8838	0.27 (0.29)	0.1817	0.33
Rey-Osterrieth Complex Figure Test immediate recall	10.94 (0.52)	12.30 (0.54)	1.44 (0.44)	0.0325	12.78 (0.40)	12.34 (0.50)	-0.60 (0.44)	0.4832	1.48 (0.63)	0.0433	0.37
Rey Auditory Verbal Learning Task delayed recall	4.02 (0.21)	4.77 (0.25)	0.72 (0.29)	0.0126	4.94 (0.24)	5.60 (0.27)	0.88 (0.28)	0.0019	0.25 (0.29)	0.7095	-0.06
Babcock short story delayed recall	2.89 (0.21)	3.70 (0.23)	0.72 (0.24)	0.0241	3.82 (0.22)	4.13 (0.22)	0.38 (0.26)	0.4253	0.22 (0.34)	0.2914	0.22
Rey-Osterrieth Complex Figure Test delayed recall	10.49 (0.52)	10.94 (0.52)	0.53 (0.42)	0.4842	12.12 (0.51)	12.55 (0.57)	0.37 (0.49)	0.5087	0.26 (0.65)	0.6899	0.01
Raven Coloured Progressive Matrices	28.27 (0.42)	28.04 (0.50)	-0.23 (0.54)	0.6705	28.37 (0.40)	28.07 (0.44)	-0.30 (0.53)	0.5690	-0.06 (0.55)	0.9271	0.02
Phonemic verbal fluency	31.63 (0.96)	34.19 (1.06)	2.48 (0.66)	0.0104	32.08 (0.86)	31.60 (1.04)	-0.50 (0.74)	0.6243	3.02 (1.03)	0.0314	0.32
Semantic verbal fluency	34.14 (0.73)	33.04 (0.82)	-0.96 (0.56)	0.1869	35.46 (0.74)	34.18 (0.83)	-1.42 (0.61)	0.1228	0.04 (0.84)	0.8815	0.02
Trail Making Test A	56.84 (3.35)	53.49 (3.74)	-3.53 (3.70)	0.4468	48.31 (2.77)	49.64 (3.03)	2.33 (2.25)	0.7572	-2.95 (4.31)	0.4493	-0.14
Trail Making Test B	169.16 (9.22)	168.97 (9.22)	0.81 (5.03)	0.9837	156.88 (9.02)	163.27 (9.55)	5.13 (7.71)	0.4970	-5.08 (9.38)	0.6199	-0.07
Trail Making Test B-A	111.64 (7.49)	110.89 (6.86)	-0.16 (5.12)	0.9295	105.76 (7.03)	114.18 (7.19)	9.15 (6.67)	0.3111	-8.08 (8.11)	0.4393	-0.12
Attentional matrices	47.76 (0.76)	47.95 (0.71)	0.21 (0.61)	0.8341	47.22 (0.75)	48.22 (0.79)	0.91 (0.62)	0.2479	0.72 (0.85)	0.5091	-0.10
Copy of Rey-Osterrieth Complex Figure test	30.05 (0.64)	29.48 (0.66)	-0.49 (0.68)	0.5366	29.60 (0.63)	30.16 (0.59)	0.18 (0.60)	0.5335	-1.02 (0.87)	0.3810	-0.17
Copy of freehand drawings	10.00 (0.13)	9.88 (0.18)	-0.16 (0.15)	0.5340	10.05 (0.15)	10.22 (0.15)	0.21 (0.14)	0.4213	-0.31 (0.20)	0.3096	-0.20
Copy of drawings with programming elements	67.75 (0.24)	66.69 (0.58)	-1.04 (0.51)	0.1004	67.72 (0.32)	66.70 (0.44)	-0.99 (0.37)	0.1138	-0.04 (0.67)	0.9642	-0.02

§: p-value from mixed-model repeated measures analyses, group*time interaction, adjusted for baseline score

Supplementary Table 3.
Baseline characteristics of subjects who were randomized to the trail, by availability of data for RM (data are expressed as n, n (%), mean±SD).

	RM available	RM not available	p-value
Intervention Group MCI-training	(n=48)	(n=7)	
Demographic characteristics			
Age at the baseline, years (mean±SD)	74.1±4.9	73.4±4.9	0.7721
Sex, women (n, %)	26 (54.2)	3 (42.9)	0.6957
Education, years (mean±SD)	9.4±4.5	9.6±5.2	0.9589
Screening Cognitive tests			
MMSE, score (mean±SD)	25.5±2.2	24.5±1.9	0.2303
CDR, n (%)<br>0<br>0.5	 1 (2.1)<br>47 (97.9)	 0 (0.0)<br>7 (100.0)	1.0000
CDT, score (mean±SD)	7.7±2.1	7.4±2.0	0.6490
Control Group MCI-no training	(n=22)	(n=36)	
Demographic characteristics			
Age at the baseline, years (mean±SD)	74.2±4.9	75.3±4.2	0.3513
Sex, women (n, %)	11 (50.0)	15 (41.7)	0.5358
Education, years (mean±SD)	8.0±3.4	9.2±4.1	0.2983
Screening Cognitive tests			
MMSE, score (mean±SD)	26.0±2.2	25.8±2.6	0.9237
CDR, n (%)<br>0<br>0.5	 1 (4.6)<br>21 (95.4)	 2 (5.6)<br>34 (94.4)	1.0000
CDT, score (mean±SD)	7.8±1.9	7.9±1.9	0.8102
